# Influence of Exogenous Factors Related to Nutritional and Hydration Strategies and Environmental Conditions on Fatigue in Endurance Sports: A Systematic Review with Meta-Analysis

**DOI:** 10.3390/nu15122700

**Published:** 2023-06-09

**Authors:** Roberto Pellicer-Caller, Raquel Vaquero-Cristóbal, Noelia González-Gálvez, Lucía Abenza-Cano, Javier Horcajo, Ricardo de la Vega-Marcos

**Affiliations:** 1 Facultad de Deporte, UCAM Universidad Católica de Murcia, 30107 Murcia, Spain; robertopellicercaller@gmail.com (R.P.-C.); ngonzalez@ucam.edu (N.G.-G.); labenza@ucam.edu (L.A.-C.); 2 Caller Energy Labs, Caller SportEnergy S.L., 39005 Santander, Spain; 3 Department of Social Psychology and Methodology, Autonomous University of Madrid, 28049 Madrid, Spain; javier.horcajo@uam.es; 4 Department of Physical Education, Sport and Human Movement, Autonomous University of Madrid, 28049 Madrid, Spain; ricardo.delavega@uam.es

**Keywords:** fatigue, endurance, exogenous factors, environmental conditions, nutrition, sport

## Abstract

The aim of this systematic review with meta-analysis was to examine the influence of exogenous factors related to nutritional and hydration strategies and environmental conditions, as modulators of fatigue, including factors associated with performance fatigability and perceived fatigability, in endurance tests lasting 45 min to 3 h. A search was carried out using four databases: PubMed, Web of Science, SPORTDiscus, and EBSCO. A total of 5103 articles were screened, with 34 included in the meta-analysis. The review was registered with PROSPERO (CRD42022327203) and adhered to the PRISMA guidelines. The study quality was evaluated according to the PEDro score and assessed using Rosenthal’s fail-safe N. Carbohydrate (CHO) intake increased the time to exhaustion (*p* < 0.001) and decreased the heart rate (HR) during the test (*p* = 0.018). Carbohydrate with protein intake (CHO + PROT) increased lactate during the test (*p* = 0.039). With respect to hydration, dehydrated individuals showed a higher rate of perceived exertion (RPE) (*p* = 0.016) and had a higher body mass loss (*p* = 0.018). In hot conditions, athletes showed significant increases in RPE (*p* < 0.001), HR (*p* < 0.001), and skin temperature (*p* = 0.002), and a decrease in the temperature gradient (*p* < 0.001) after the test. No differences were found when athletes were subjected to altitude or cold conditions. In conclusion, the results revealed that exogenous factors, such as nutritional and hydration strategies, as well as environmental conditions, affected fatigue in endurance sports, including factors associated with performance fatigability and perceived fatigability.

## 1. Introduction

Fatigue can be defined as a disabling symptom in which physical and cognitive functions are limited by interactions between performance fatigability, defined as the decline in an objective measure of performance over a discrete period, and perceived fatigability, defined as changes in the sensations that regulate the integrity of the performer. Specifically, performance fatigability depends on the contractile capabilities of the muscles involved, and the capacity of the nervous system to provide an adequate activation signal, while perceived fatigability depends on the psychological state and feelings that regulate the integrity of the performer, which are based on the maintenance of homeostasis [1,2].

Fatigue has been widely studied in endurance sports that last 45 min to 3 h [3,4,5,6,7,8,9,10,11,12,13,14,15,16,17,18,19,20,21,22,23,24,25], due to the high number of sports with this duration. More specifically, both in acyclic sports, such as soccer, football, hockey, etc., and in cyclic sports, such as marathons, triathlons, cross-country skiing, etc., the duration of the training and competition usually falls within this time frame. In addition, it is common for tests that are used to evaluate the performance and thresholds of athletes in these sports to also be of this duration [26,27]. Above this length of time, according to previous studies, one would enter another type of sporting modality, defined as ultra-endurance sports [28], in which fatigue could be affected by other performance and perceived fatigability factors [29,30].

In endurance sports, fatigue makes it impossible to maintain the intensity required, due to a combination of factors related to perceived fatigability and performance fatigability [1,2,31]. Thus, factors such as blood glucose availability, body and external temperature, the presence of metabolites, previous hydration status and fluid intake during exercise, oxygenation, and psychological state, could affect perceived fatigability, while calcium kinetics, force capacity, blood flow, metabolism, and products or factors related to muscle activation, could affect performance fatigability, limiting performance in this type of sports [1,2,3,4,5,6,7,8,9,10,11,12,13,14,15,16,17,18,19,20,21,22,23,24,25,31].

More specifically, many authors have shown that in these sports, fatigue is associated, among other factors, with the body’s inability to sustain the production of energy through the active metabolic pathway, resulting in decreased performance [32], the generation of physiological and metabolic changes, such as hypoglycemia [32], hyperammonemia [33], glycogen depletion [34], hydro-electric disturbances [3,4], and poorer thermoregulation [5,6]. These factors affect the onset of fatigue from a full-scale point of view, as they not only affect the contractile function and muscle activation parameters (i.e., related to performance fatigability), but they can also affect homeostasis and the psychological state (i.e., perceived fatigability) [1,2].

The occurrence of factors associated with sports fatigue could be delayed by using different nutritional strategies, such as exogenous factors [35]. Hypoglycemia can occur due to glycogen depletion, which limits the muscles involved [34], leading to fatigue. Due to glycogen depletion and glucose oxidation deficits, fatty acid oxidation becomes the main source of energy. This pathway is slower, given the difficulty in diffusing fatty acids through the capillaries to the mitochondria, thereby making beta-oxidation a high capacity, low-power process [32]. This is why hypoglycemia is treated in the process of fatigue, through strategies used to modify energy reserves: replenishing glycogen and administering CHO during the practice of sports, with both strategies lengthening the time until exhaustion [7,8,9,10,11,12,13,14,15,16,17,24,25].

Hyperammonemia occurs when glycogen is low, indicating that glycolysis is limited and cannot meet the demands of the exercise [36]. As a result, the body increases the energy obtained through other substrates, such as protein catabolism, although it can increase blood ammonium concentration [37], and trigger fatigue, especially at the central level [33]. Thus, protein intake during the test could be another strategy that could be used to delay the onset of fatigue [18,19,20,21,22,23].

Electrolyte deficiency and poorer thermoregulation may also be a cause of fatigue [3,4]. The most studied electrolyte is potassium, which can decrease in muscles due to losses during exercise, although these losses are minimal and should not influence fatigue [4]. Another electrolyte, magnesium, increases in concentration during exercise, and inhibits the release of muscular calcium, decreasing muscular contraction [3]. In addition, hydroelectric disturbances can be induced by changes in hydration. A hydroelectric imbalance can alter homeostasis, which can affect, among other factors, physiological function and exercise performance [38,39,40,41]. Therefore, hydration before and during the test can delay fatigue [42,43,44].

Nevertheless, there are also certain environmental factors that exogenously influence fatigue. With regard to heat, it has been verified that a core body temperature over 40 °C, which could be induced by poor thermoregulation and high outside temperature, accelerates the onset of fatigue from an all-encompassing point of view, with factors associated with performance fatigability and perceived fatigability in particular [45], producing an increase in the heart rate (HR) and the rate of perceived exertion (RPE), respectively [46,47,48,49,50]. In relation to cold, it decreases oxygen consumption (VO_2_) due to a decrease in the HR and cardiac output [51]. At altitude or hypoxia, the body attempts to compensate for the increased partial pressure of oxygen in the arterial blood (PaO_2_) [52], resulting in increased ventilation, HR [53] and VO_2_, accelerating the onset of fatigue and decreasing performance [53].

However, to the best of our knowledge, no systematic review or meta-analysis was found that analyzed the influence of exogenous factors related to nutritional and hydration strategies and environmental conditions, as modulators of performance and perceived fatigability factors in sports lasting from 45 min to 3 h, despite the fact that there are many cyclic and acyclic sports in which the duration of training and competition falls within this time frame [26,27]. In addition, on many occasions the limitation to continue comes as a result of the accumulation of fatigue [32], although the performance and perceived fatigability factors associated with fatigue may be different from longer duration sports, which are considered ultra-endurance sports [28,29,30]. Therefore, the aim of this systematic review with meta-analysis was to examine the influence of exogenous factors, related to nutritional and hydration strategies and environmental conditions, as modulators of fatigue, including factors related to both performance fatigability and perceived fatigability, in endurance tests lasting between 45 min to 3 h.

## 2. Materials and Methods

### 2.1. Design

The studies included in the present systematic review with meta-analysis examined the influence of exogenous aspects related to nutrition and hydration, as well as environmental conditions, on performance and perceived fatigability aspects in tests lasting between 45 min and 3 h. The search strategy, inclusion criteria, and additional information were previously registered with the international prospective systematic review register, PROSPERO (CRD42022327203) (direct link: https://www.crd.york.ac.uk/prospero/display_record.php?RecordID=327203, accessed on 31 May 2023). This meta-analysis adhered to the preferred reporting items for systematic reviews and meta-analyses (PRISMA) guidelines [54,55].

### 2.2. Search Strategies

Four electronic databases were searched until 15 June 2022: PubMed, Web of Science, SPORTDiscus, and EBSCO. The search strategy was created for Web of Science and adapted to the PubMed, SPORTDiscus, and EBSCO databases. The reference lists for the articles included in the study were manually searched for relevant additional studies. The search formula used is provided in Supplementary S1.

The inclusion criteria were: (1) experimental or quasi-experimental studies, with randomized controlled designs; (2) containing a control and experimental group, or experimental groups only; (3) determining factors associated with fatigue in endurance sports from 45 min to 3 h; (4) analyzing the acute effect of performance or perceived fatigability; (5) including healthy participants aged 18–65 years; (6) making exogenous modifications to observe their effects on performance or perceived fatigability factors, more specifically, studies in which athletes in the experimental group ingested CHO, CHO + PROT or liquids during the test, or were subjected to conditions of altitude, cold or heat; (7) the written language was English, Spanish, Italian, or Portuguese; and (8) no time limit. The exclusion criteria were: (1) short communication, note, letter, review article or brief report; (2) not having used any exogenous factors related to nutritional and hydration strategies and environmental conditions as an independent variable to try to modify the onset of fatigue; and (3) not having at least three papers that analyzed the influence of the same independent variable on the same performance or perceived fatigability dependent variable.

### 2.3. Data Extraction

The search was conducted independently by two reviewers (RP-C. and RV-C.), who examined the titles and abstracts of the articles in the first screening, and the full texts in the second screening, to determine which articles should be included in the meta-analysis. A third reviewer (RV-M.) was consulted to resolve any disagreements about the inclusion of the articles. To determine inter-reviewer reliability, Cohen’s kappa (45) was used, which showed a strong level of agreement (kappa = 0.914).

### 2.4. Quality Assessment and Risk of Bias

To assess the quality of the included studies, the PEDro scale was used. Two reviewers (RP-C. and RV-C.) were responsible for the quality assessment of the studies. A third reviewer was consulted to resolve any disagreements (LA-C.). Rosenthal’s [56] fail-safe N was used to assess the risk of bias. Egger’s test to detect bias is limited when the number of studies is small (i.e., less than ten) [57]. Therefore, this test must be performed when there are at least ten studies included in the meta-analysis [58]. Thus, neither Egger’s test nor funnel plots were performed or created [58].

### 2.5. Data Analysis

The statistical analysis and meta-analysis were performed using the Comprehensive Meta-Analysis software program (version 3, Englewood, Bergen County, NJ, USA) [59]. The meta-analysis was performed for continuous data using the mean and standard deviation of each variable for the different measurements taken. This information was extracted directly from the studies. The analysis was performed when at least three studies analyzed the same dependent variable. For studies that did not present the necessary data, standard deviations (SDs) were calculated and imputed where possible, using standard errors (SEs) and confidence intervals (CIs). We used the DerSimonian–Laird (Cohen) clustering method and assessed heterogeneity, using the Cochrane Q test (Chi^2^), Higgins *I*^2^, and significance (p) [60]. A meta-analysis with a random-effects model was performed to infer the pooled estimated standardized mean difference (SMD) [61]. The pooled effect was obtained using the inverse of variance method. The DerSimonian–Laird (Cohen’s) SMD was interpreted using Cohen’s D [62], defined as small (0 to 0.2), medium (0.3 to 0.7), and large (≥0.8).

## 3. Results

### 3.1. Data Search and Study Characteristics

**Figure 1:**
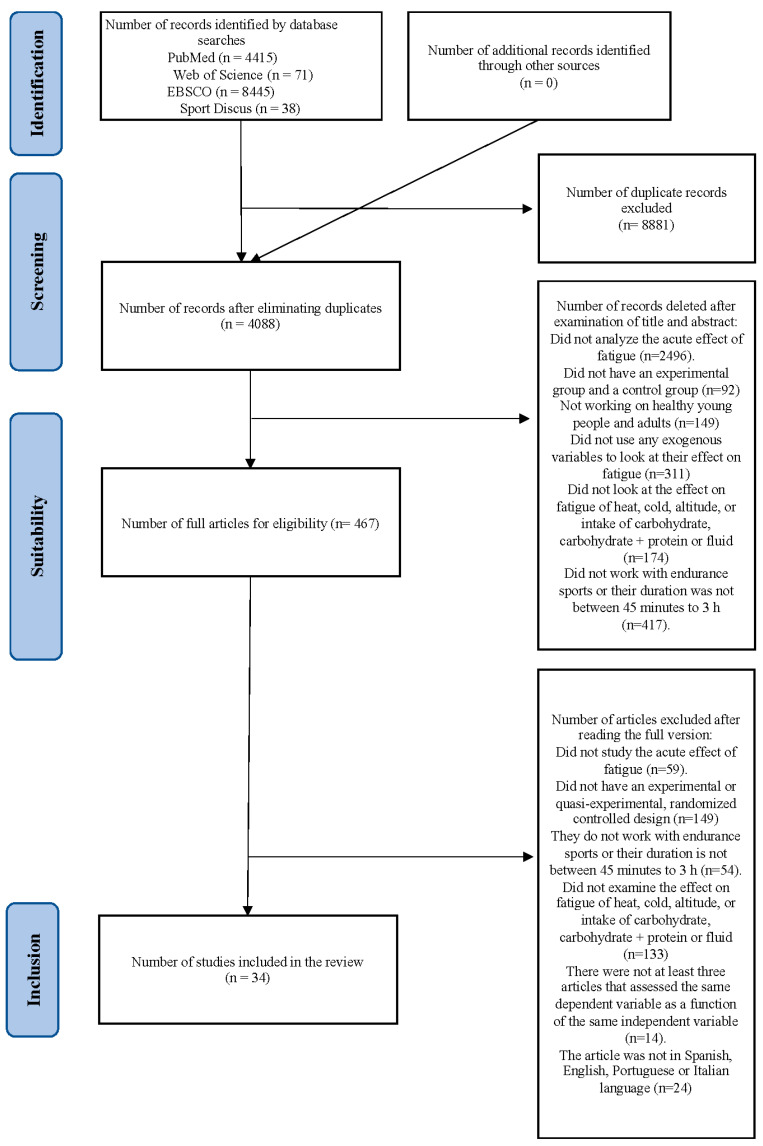
Flow diagram of the studies searched, screened, and included in the meta-analysis.

Thirty-four articles were included in the systematic review with meta-analysis (Figure 1). Among all the studies, a total of 464 participants were included in the experimental groups and 472 participants in the control groups. Participants were aged 18–46 years old in the experimental groups and 18–46 years old in the control groups.

#### 3.1.1. Influence of Nutritional and Hydration Strategies to Modify Performance and Perceived Fatigability

According to the modified exogenous conditions (Table 1), 23 studies analyzed aspects related to nutrition or hydration as an independent variable with which to modify the onset of fatigue. More specifically, 11 studies analyzed CHO during the test [7,8,9,10,11,12,13,14,15,16,17], and included 109 participants in the experimental groups and 109 participants in the control groups. Of the 11 studies, 7 were on males [7,9,12,14,15,16,17] and 4 on both sexes [8,10,11,13]. Six studies analyzed CHO + PROT during the tests [18,19,20,21,22,23], including 74 participants in the experimental groups and 74 participants in the control groups, with all participants being males [18,19,20,21,22,23]. Six studies studied hydration status during the tests [38,39,40,42,43,44], including 176 participants in the experimental groups and 184 participants in the control groups, with 5 of them including only males [38,40,43,44] and 1 with both sexes [39].

Regarding the characteristics of the sample included in the different studies (Table 1), those focused on CHO during the tests included trained cyclists (*n* = 4) [7,10,15,16], runners (*n* = 2) [9,11], tennis players (*n* = 2) [12,14], soldiers (*n* = 1) [17], and physical active/recreational athletes (*n* = 2) [8,13]. The CHO + PROT intake studies included trained cyclists (*n* = 2) [18,21], healthy runners [20], and healthy trained individuals (*n* = 3) [19,20,21,22,23]. The studies that analyzed hydration included basketball players (*n* = 1) [38], trained cyclists (*n* = 3) [39,40,43], hockey players (*n* = 1) [42], and recreationally active individuals (*n* = 1) [44].

Regarding the test performed (Table 1), in relation to CHO intake during the test [7,8,9,10,11,12,13,14,15,16,17], it was found that one study worked with a 110% anaerobic threshold until exhaustion [7], one study worked with 120–130% VO_2_max intervals and rest until exhaustion [8], four studies worked with a constant 70–90% VO_2_max until exhaustion [9,10,11,15], two studies simulated a strenuous tennis match [12,14], one study worked with a time trial at maximum performance ending with a Wingate test [16], one study worked with two sessions of directed interval classes [13], and one study worked with a loaded, strenuous walk at a constant speed of 4.4 km/h [17]. In relation to CHO + PROT intake during the test [18,19,20,21,22,23], one study worked with a time trial at maximum performance [18], three studies worked with a steady 70–75% VO_2_max until exhaustion [19,20,21], one study worked with two intensities, namely 80% of respiratory compensation point and 110% of maximum power, until exhaustion [22], and one study worked with 10 min at 50% VO_2_max plus a 30 min interval at maximum intensity [23]. Of the studies that analyzed hydration during the test [38,39,40,42,43,44], one study was found to work at 50% VO_2_max plus a simulated match [38], one study performed an endurance cycling competition at its own pace [39], and one study worked at a 95% lactate threshold plus a time trial at maximum intensity [40], one study worked with a time trial at maximum intensity [43], one study worked with a simulated hockey match [42], and one study worked at constant 60% of the VO_2_max [44].

Regarding the exogenous conditions to which the participants in the experimental group were subjected (Table 1), in relation to CHO during the test, it was found that five studies examined the ingestion/mouth rinse of 6–8% CHO during the test [9,10,13,14,16], one study used an ingestion of 18% CHO before the test and 6% CHO during the test [8], and four studied an intake of 6–8% CHO and electrolytes during the test [7,11,15,17]. In relation to the studies that used a combined intake of CHO + PROT, one looked at an intake of 6–8% CHO and 1.5–2% PROT during the test [18,21,22], in one study, the participants ingested amino acids (BCAA) with CHO [19], in another, the participants ingested 1.2 g/kg/h CHO + 0.4 g/kg/h hydrolyzed whey protein [20], and, lastly, in one study, the participants ingested tryptophan + CHO [23]. On hydration, in three studies, the participants who performed the test were euhydrated in environmental conditions that were thermoneutral (23 °C) [39,40] or with a temperature of 40 °C [38]. In one study, the participants ingested only water at a rate of 26 mL/kg^−1^ [43], in another, participants ingested a drink containing CHO + electrolytes [42], and, in the last study, participants were hydrated with water ad libitum [44].

Finally, regarding the dependent variables included in the studies (Table 1), within the studies that analyzed CHO intake during the tests [7,8,9,10,11,12,13,14,15,16,17], five assessed time until exhaustion [7,8,9,10,17], three measured lactate [9,14,17], and four measured HR [14,15,16,17]. As for CHO + PROT intake during the tests [18,19,20,21,22,23], four measured lactate [18,19,20,21], three measured RPE [21,22,23], and four measured HR [19,20,21,23]. On hydration during the test, three measured RPE [38,39,40], and three measured body mass loss in kilograms [42,43,44].

#### 3.1.2. Influence of Environmental Conditions to Modify Performance and Perceived Fatigability

Our systematic review found eleven studies that examined the influence of exposure to environmental factors as an exogenous factor, on the occurrence of fatigue. More specifically, three studies analyzed the altitude effects during the test [63,64,65], including 50 males in the experimental groups and 50 males in the control groups. Three studies looked at cold exposure during the test [66,67,68], including 33 participants in the experimental groups and 33 participants in the control groups, with all of them being male [66,67,68]. Five studies examined heat exposure during the test [46,47,48,49,50], including 55 participants in the experimental groups and 55 participants in the control groups. Four of these studies included only males [46,48,49,50] and one included both sexes [47].

Regarding the characteristics of the sample included in the different studies (Table 2), the studies on altitude effects included trained cyclists (*n* = 1) [63] and healthy trained/physically active adults [64,65]. The studies examining the effects of cold temperatures included acclimatized healthy active people (*n* = 1) [66], acclimatized healthy cyclists (*n* = 1) [67], and active healthy people (*n* = 1) [68]. Lastly, the studies on heat effects included trained and untrained active healthy participants (*n* = 2) [47,50], and acclimatized and non-acclimatized trained cyclists (*n* = 3) [46,48,49].

Regarding the test performed (Table 2), in relation to altitude conditions [63,64,65], one study was found to work with a 70% VO_2_max until exhaustion [63], one study worked at 50% VO_2_max plus 15 min at a non-constant, maximal speed subject to the participant’s self-perception [64], and one study worked at a constant HR of 140 beats/minute [65]. In relation to cold during the test [66,67,68], two studies worked at 75–80% VO_2_max until exhaustion [66,67,68], and one study worked with a time trial at maximum intensity [67]. Of the studies that looked at heat conditions [46,47,48,49,50], we found one study that worked with a time trial at maximum intensity [46], one study worked with a treadmill walk at a speed of 4.5 km/h with a 2% incline [47], and three studies worked at 60% VO_2_max until exhaustion [48,49,50].

Regarding the exogenous environmental conditions to which the participants of the experimental group were subjected (Table 2), three studies used an altitude between 2000 and 3000 m [63,64,65]. Regarding cold conditions, two studies had the participants perform exercise at temperatures between 0 and 5 °C [67,68], while another study had them perform exercises in cold conditions using a head cooling strategy to lower the core temperature [66]. In relation to heat conditions during the test, five studies had the athletes perform exercise at 35–40 °C [46,47,48,49,50].

Finally, regarding the dependent variables included in the studies (Table 2), within the studies that analyzed exposure to altitude during the test, three measured RPE [63,64,65], and three measured HR [63,64,65]. Under cold conditions during the test, three measured lactate [66,67,68]. Finally, under hot conditions during the test, three measured RPE [46,47,48], four measured HR [46,48,49,50], two measured skin temperature (°C) [48,50], and two measured temperature gradient (°C) [48,50].

### 3.2. Quality Assessment and Publication Bias

The results obtained with the PEDro scale (Table 3) showed that studies that used nutritional and hydration strategies obtained scores between 6 and 11, indicating good to excellent methodological quality. More specifically, for the studies that analyzed CHO intake during the test, 8 scored between 9 and 11, obtaining excellent methodological quality; and three scored between 6 and 8, with a good quality. With respect to the studies that conducted intakes of CHO + PRO during the test, six scored between 9 and 11, with excellent methodological quality. For hydration, one obtained a score of 11, obtaining an excellent methodological quality, while another five obtained a score between 6 and 8, indicating a good quality.

Somewhat more disparate scores were found in the studies that analyzed the effects of environmental conditions as exogenous factors, on variables related to performance and perceived fatigability (scores from 4 to 11, with fair/good/ excellent methodological quality). More specifically, with respect to altitude studies, one obtained a score of 9, indicating excellent methodological quality, while two obtained a score between 6 and 7, indicating good quality. For cold temperatures during the test studies, one received a score of 11, indicating excellent quality, with another two studies receiving scores between 4 and 5, indicating a fair quality. Lastly, for hot conditions during the test studies, three obtained a score of 6, indicating good quality, while the other two studies obtained a score of 5, indicating fair quality.

### 3.3. Results Found with the Modification of Exogenous Variables Related to Nutrition and Hydration

**Figure 2:**
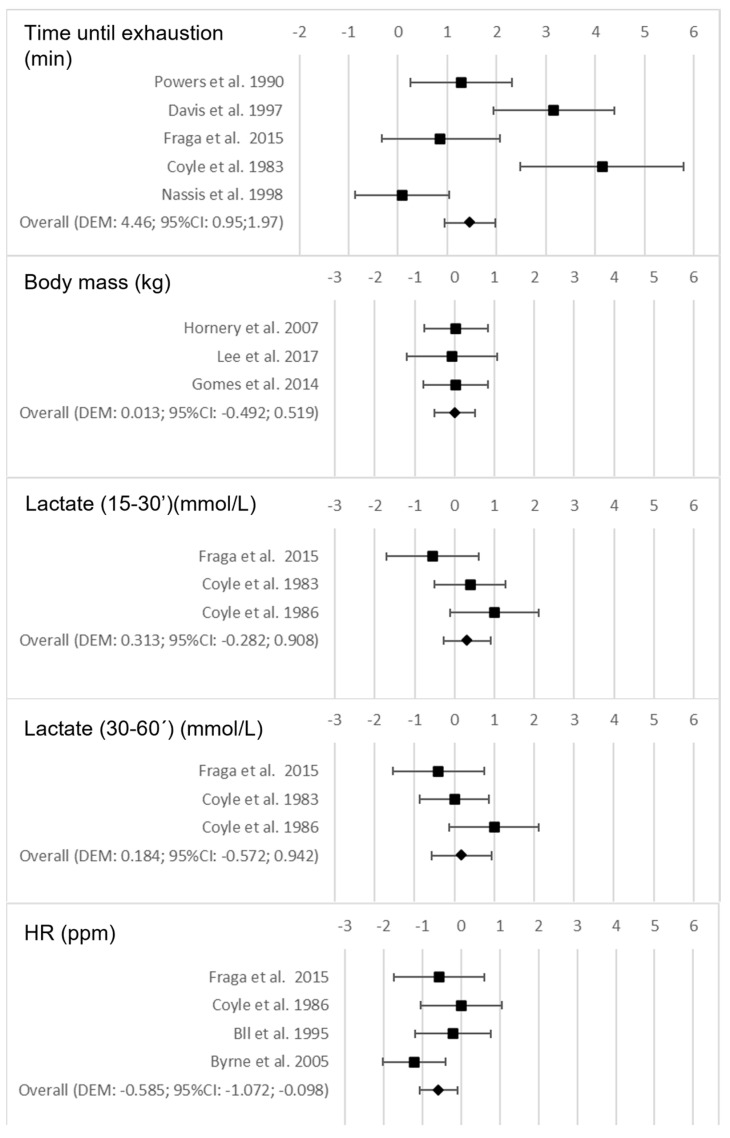
Forest plots on the influence of the intake of carbohydrates (CHO) on the performance and perceived fatigability variables during the test [7,8,9,10,11,12,13,14,15,16,17].

The changes in performance and perceived fatigability resulting from the use of nutritional and hydration strategies are shown in Table 4. Importantly, it was found that CHO intake during the test increased the time until exhaustion when comparing the results of the experimental group (EG) with the control group (CG) (*p* < 0.001). In addition, the increase in HR during the test was lower in the EG than in the CG (*p* = 0.018). In contrast, no differences were found between the EG and CG for body mass loss during the test (*p* = 0.959), or in lactate accumulation during the test, at none of the times measured (min 15′–30′ or 30′–60′) (*p* = 0.303 and *p* = 0.633, respectively). Figure 2 shows the forest plots for the variables that were examined as a consequence of CHO intake between the EG and the CG.

**Figure 3:**
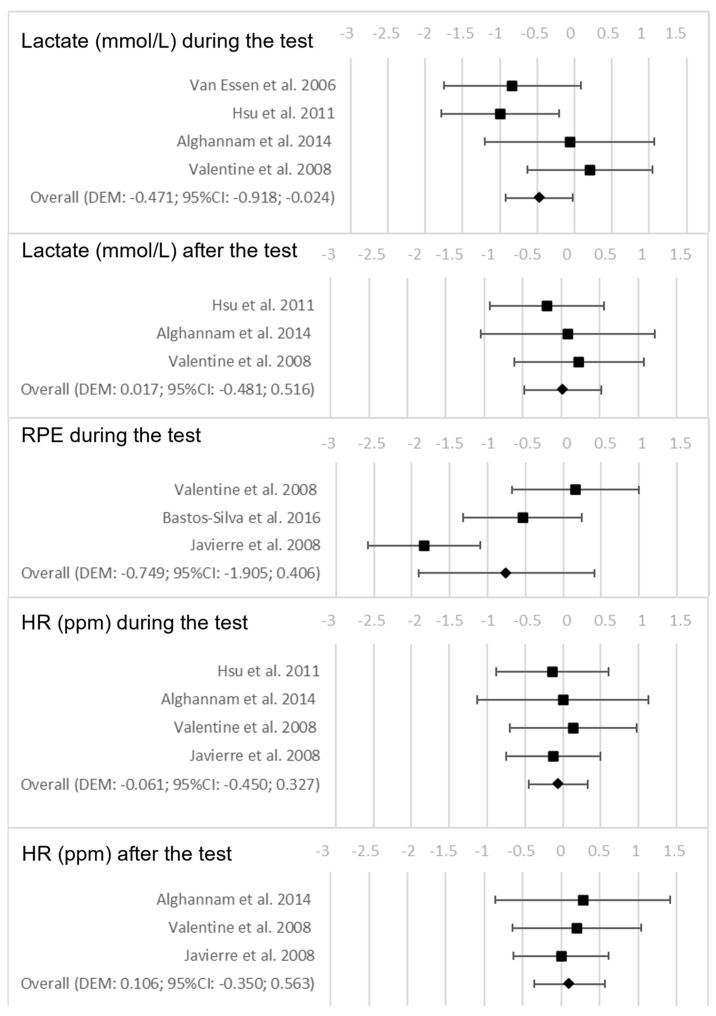
Forest plots on the influence of the intake of carbohydrates + proteins (CHO + PROT) during the test on the performance and perceived fatigability variables during the test and after the test [18,19,20,21,22,23].

Regarding CHO + PROT intake during the test, a lower lactate accumulation during the test was found in the EG than in the CG (*p* = 0.039). However, there were no significant differences in the lactate accumulation after the test (*p* = 0.946), nor differences in the RPE during the test (*p* = 0.204), and the HR during (*p* = 0.756) or after the test (*p* = 0.647). Figure 3 shows the forest plots for the variables that were examined as a consequence of CHO + PROT intake between the EG and the CG during and after the test.

**Figure 4:**
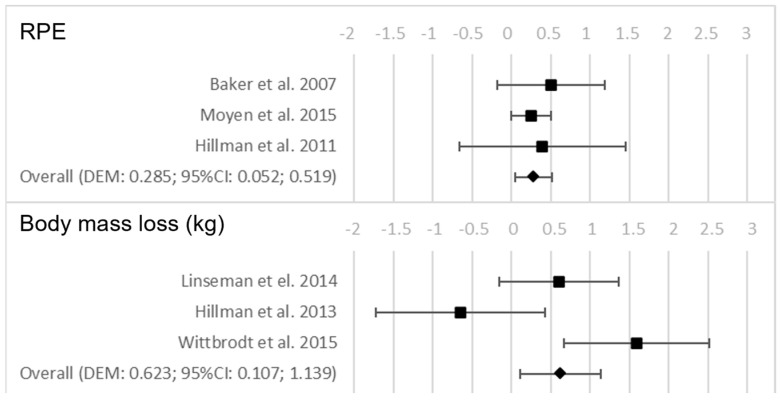
Forest plots on the influence of hydration on the performance and perceived fatigability variables after the test [38,39,40,41,42,43,44].

In relation to hydration, it was found that the groups that were dehydrated during the test showed a significantly higher RPE (*p* = 0.016). In addition, those groups that did not hydrate during the test had a significantly higher body mass loss in kg (*p* = 0.018). Figure 4 shows the forest plots for the variables that were examined as a consequence of the hydration conditions after the test.

### 3.4. Results Found with the Modification of Exogenous Variables Related to Environmental Conditions

**Figure 5:**
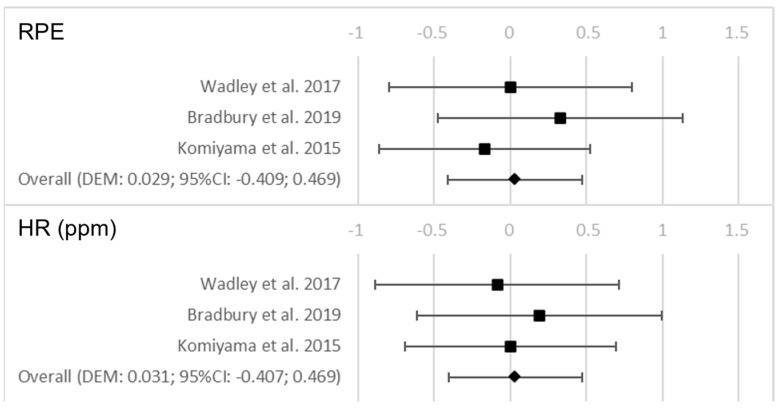
Forest plots on the influence of the altitude conditions on the performance and perceived fatigability variables after the test [63,64,65].

The changes in performance and perceived fatigability as a result of exposure to different environmental situations are shown in Table 5. With respect to high altitude conditions, the results indicated no significant differences for either the RPE (*p* = 0.894) or HR after the post-test analysis (*p* = 0.890). Figure 5 shows the forest plots for the variables that were examined as a consequence of the altitude conditions after the test.

**Figure 6:**
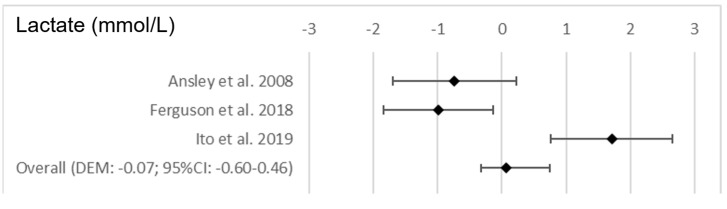
Forest plots on the influence of the cold conditions on the performance and perceived fatigability variables after the test [66,67,68].

In relation to the performance in cold conditions, no significant change was found in the post-test lactate (*p* = 0.790). Figure 6 shows the forest plots for the variables that were examined as a consequence of the cold conditions after the test.

**Figure 7:**
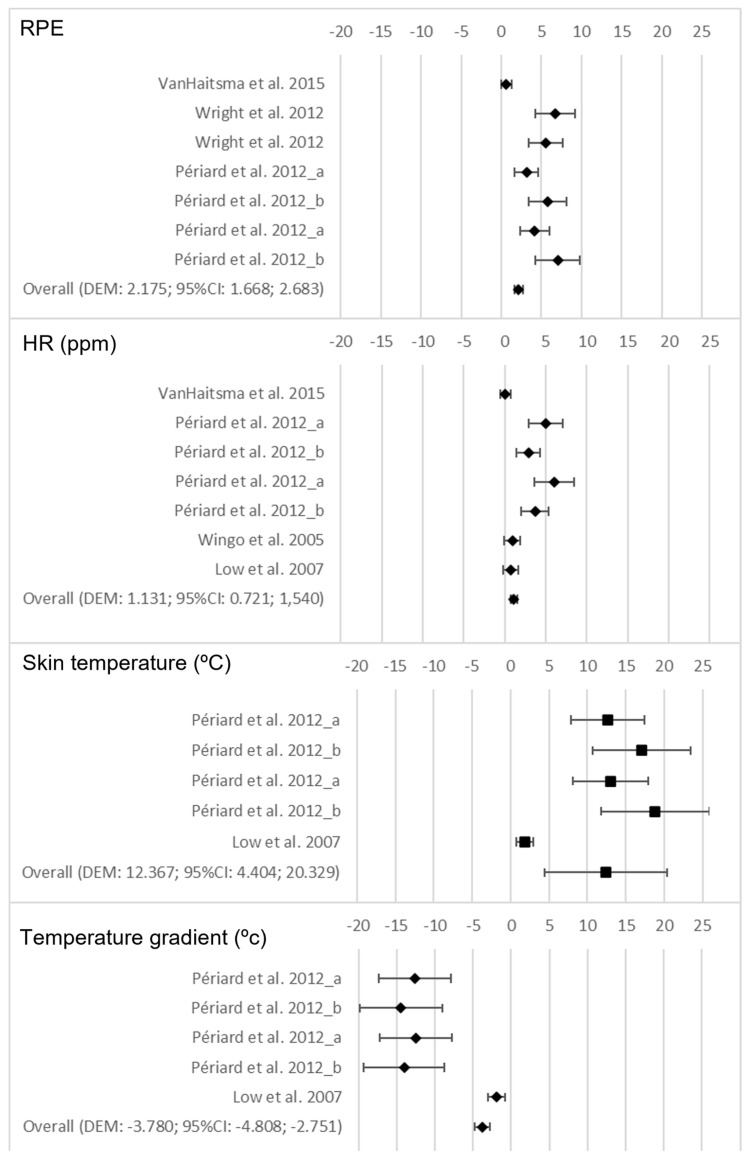
Forest plots on the influence of the hot conditions on the performance and perceived fatigability variables after the test [46,47,48,49,50].

Regarding the exposure to hot conditions, it was found that, as compared to the CG, the EG showed significantly higher values for RPE (*p* < 0.001), HR (*p* < 0.001), and skin temperature (0.002) after the test, as well as significantly lower values for the temperature gradient (*p* < 0.001). Figure 7 shows the forest plots for the variables that were examined as a consequence of the hot conditions.

## 4. Discussion

### 4.1. Influence of CHO Intake on Fatigue

This systematic review with meta-analysis found that a CHO intake of 6–8% during an endurance test increased the time until exhaustion [7,8,9,10,17]. This could be because the intake of CHO increases glycemia in blood, delaying muscular and hepatic glycogen depletion [25,69,70], as it increases the amount of glycogen available as an energy source [69,70], thus increasing the time until exhaustion [7,8,9,10,17,71]. In fact, it has been demonstrated that exhaustion coincided with an almost complete glycogen depletion [69,70,72], and the greater the amount of glucose in the blood, the greater the delay to exhaustion [25,69,70].

Another relevant finding in the present systematic review with meta-analysis was that CHO intake during the test decreased the HR during the test. This could be because, as shown in previous studies, the availability of CHO delays overexertion in different systems by achieving energy maintenance and lower metabolic stress and performance fatigability [73]. On the contrary, low glucose availability leads to increased HR because of the increased metabolic demands, anticipating the occurrence of fatigue [69,70,73].

In contrast, the present systematic review with meta-analysis found that CHO intake did not have an influence on the loss of body mass assessed after the maximal test. This finding is in contrast with previous studies, which argued that CHO availability increases the time until exhaustion, and as a consequence of sustaining exertion for a longer period of time, body mass loss may also be greater, mainly as a consequence of fluid loss through sweating [12,13,14]. However, it has to be taken into consideration that fluid intake during the test may be the most important factor in body mass loss, over and above the length of time the test is maintained [45,74,75]. Therefore, given these findings, the effects of CHO intake during the test on body mass, as a function of the athlete’s fluid intake during the test, should be considered in future studies.

Another relevant finding was that the intake of CHO during the test did not influence lactate accumulation [9,14,17]. As previous studies have indicated, intense lactate increases in trained subjects can occur from 80% of maximal aerobic capacity or even above the anaerobic threshold in some cases [76]. However, in the case of the studies included in the present review, most of them proposed a continuous test and tests below the anaerobic threshold. Thus, energy generation via the anaerobic pathway was practically nil, with lactate accumulation not being a limiting or differential factor when subjecting the participants to exogenous constraints [77].

### 4.2. Influence of CHO + PROT Intake on Fatigue

An interesting finding in the present review was that the athletes who consumed a combination of CHO and protein during the endurance test showed a lower increase in lactate during the test [18,19,20,21]. There has been much debate about whether protein intake along with CHO is necessary to improve sports performance or decrease the incidence of fatigue in endurance sports [18,19,20,21]. This is especially true when the amount of CHO ingested is near optimal for exogenous carbohydrate oxidation [78], even indicating that in these cases protein does not improve performance during a task that closely simulates athletic competition [18]. Therefore, it is possible that in the studies that combined CHO + PROT, CHO intake was the factor that most affected the delay of fatigue, given its influence on the availability of glucose in the blood, which could delay the depletion of energy reserves [73,79,80]. This, coupled with the fact that most studies presented continuous exercise protocols below the anaerobic threshold, could explain the delay in the main role of the anaerobic metabolic pathway as the main route for obtaining energy to maintain the effort [73,79,80], which would explain why in the intermediate phases of the test there was a lower accumulation of lactate in the group that consumed CHO + PROT. In fact, there were no differences between the groups after the test, which would support the hypothesis that the activation of the lactic anaerobic pathway as the main energy source was delayed in the face of the continued use of the aerobic metabolic pathway [73,79,80]. However, the different studies presented different ratios of CHO + PROT, with studies above and below maximal rates of exogenous carbohydrate oxidation, making it difficult to determine whether improvements in endurance were related to variations in total energy intake or the specific result of the CHO and/or protein mediation effects [21]. Given these interesting preliminary results, it would be advisable to analyze the evolution of the lactate curve throughout the test in future research, with different proportions of CHO + PROT or CHO alone, but matching the caloric intake of the formulas used.

Another important finding from this review was that the consumption of CHO + PROT during the test resulted in no changes in the RPE during the test and no changes in the HR during and after the test [19,20,21,23]. This could be due to the fact that in most of the included studies, the CHO loads were below 6%, which may be the limiting value for obtaining a significant effect on the RPE and HR [81]. Despite these interesting results, some questions remain pending and future research should examine them.

### 4.3. Influence of Hydration on Fatigue

The results from the present systematic review with meta-analysis indicated that participants who were dehydrated or did not hydrate during the test showed a higher RPE and body mass loss after an endurance test [38,39,40]. The absence of adequate fluid replacement during a long duration event could lead to a loss of body mass as a consequence of body fluid loss [42,43,44]. Indeed, previous studies have shown that the change in body mass accurately reflects the change in total body water after prolonged exercise [82]. However, the association between this factor and sporting performance is not clear, as previous studies have found that faster athletes present the highest body mass lost, perhaps as a consequence of the exercise’s physiological demands associated with a higher intensity or a longer duration of effort [83,84]; further studies are needed to analyze the direct or indirect influence of this aspect on performance and perceived fatigue. In addition, previous studies have pointed out that during states of dehydration, there is a decrease in cutaneous blood flow, which hinders thermoregulation, leading to an increase in core temperature, a factor highly associated with the appearance of perceived fatigability and an increased RPE [45,81].

### 4.4. Influence of High-Altitude Environments on Fatigue

The present systematic review with meta-analysis showed that at altitudes ranging from 2000 to 3000 m above sea level, no changes were observed in the RPE or HR relative to the sea level after an endurance test [63,64,65]. The explanation could be that the athletes may have acclimatized to the altitude [85], after which most physiological parameters behave in a similar way as when the athlete is at sea level [86,87]. Given these preliminary results, it would be interesting in future research to analyze whether there are differences in the evolution of performance and perceived fatigability when performing a maximal test according to the number of days the athlete spends at altitude, or according to the altitude to which they are subjected.

### 4.5. Influence of Cold Environments on Fatigue

Regarding the studies that analyzed the effects of testing in cold temperatures, between 0 and 5 °C [67,68], and cold plus head cooling [66], there was no change in lactate at the end of the test [66,67,68]. Previous studies have indicated that cold temperatures lead to an increased metabolism of CHO and lipids, among others, with increased glucose oxidation, and glycogen and lactate utilization, which maintain or decrease the amount of lactate in muscles [88]. Therefore, intramuscular lactate exhaustion is higher or the same in cold conditions, due to the increase in energy requirements when facing a decrease in external temperature [89]. However, the effect of cold environments on the other performance and perceived fatigability parameters needs to be further studied in future research.

### 4.6. Influence of Hot Environments on Fatigue

The present systematic review with meta-analysis showed that in conditions of heat with temperatures between 35 and 40 °C, higher values were found in skin temperature increases at the end of an endurance test, while the temperature gradient decreased significantly [48,50]. Furthermore, the HR [46,48,49,50] and RPE [46,47,48] also increased at the end of the test. Previous studies have found that in hot environments, it is more difficult to dissipate heat through conduction, convention, or radiation, which necessitates a greater involvement of evaporation as a means of thermoregulation, leading to peripheral vasodilation and increased sweating [90]. Consequently, there is a transfer of blood to the skin, and a decrease in blood flow to non-involved internal areas [91]. In addition, there is a decrease in venous return due to increased sweating, and during this process, there is also an increase in the HR in an attempt to maintain cardiac output despite the decrease in stroke volume [45,81]. The maintenance of exercise under these circumstances, with a continued loss of blood volume, leads to an increasing inability to dissipate heat [48,50], resulting in the temperature changes found in this systematic review with meta-analysis. All of these factors have been associated with RPE and both performance and perceived fatigability [92].

### 4.7. Limitations

The studies included in this systematic review with meta-analysis are not without limitations. These include the samples included, with few studies involving women, and the common use of small samples. Another limitation is the great heterogeneity of the sample in terms of the condition of the athletes, competitive level, modality practices, etc. There was also a lack of consensus on the protocol used to bring participants to exhaustion. Previous studies have found that factors such as sporting background, sport played, competitive level, the characteristics of the endurance test performed, or gender, could affect performance and perceived fatigability [93,94,95,96]. Therefore, more research is needed in this regard. Another limitation is the lack of inclusion in most studies, of sufficient variables to analyze fatigue from a multifactorial point of view. A final limitation is the presence of exogenous factors for which there is virtually no research. These limitations could be future lines of research.

Aside from the abovementioned research-related limitations, other database limitations were found. Among them, we note that some databases, such as Scopus, Applied Social Sciences Index and Abstracts (ASSIA), PsycINFO, Virtual Health Library, CINAHL, and Cochrane Library, among others, were not included in the search. However, the main sport science and sports nutrition databases were chosen, following the example in previous research [97]. Another limitation to this systematic review with meta-analysis is that it did not analyze the effect of other exogenous factors, such as buffering agents. More specifically, factors such as sodium bicarbonate, β-alanine, sodium citrate, sodium, and calcium lactate, among others, have been proposed as agents that could have an impact on performance and perceived fatigability during endurance tasks, with counter-argumentative results [98,99,100,101,102,103]. However, this was due to the fact that after applying the inclusion and exclusion criteria, at least three articles were not available that analyzed the influence of the same buffering agents on the same performance or perceived fatigability variable. Therefore, more research is needed on their use in endurance tests lasting between 45 min and 3 h.

### 4.8. Practical and Theoretical Applications

As practical applications of the present systematic review with meta-analysis, it was found that CHO or CHO + PROT intake, together with an adequate hydration strategy during an endurance test, may be essential for delaying the onset of fatigue, as indicated regarding performance and perceived fatigability. This may be because increased blood glucose levels during endurance events may increase the availability of glycolysis as the main source of energy, delaying the depletion of muscle and liver glycogen stores and the emergence of the anaerobic metabolic pathway as the main source of energy. This, in turn, leads to lower metabolic stress, which could affect both performance and perceived fatigability variables. Furthermore, inadequate fluid replenishment may affect thermoregulation, with affects both performance and perceived fatigability variables. This is an issue that should be considered by sport nutritionists and athletes involved in competitions/training sessions lasting between 45 min and 3 h, especially when performing sports in hot conditions, as this is a determining factor in the appearance of performance and perceived fatigability, which could be due to the fact that in these environmental conditions, heat dissipation becomes more challenging, requiring increased evaporation for thermoregulation, at the same time that blood volume loss in hot conditions impairs heat dissipation and contributes to fatigue.

Regarding the theoretical implications, it is still unclear which ratio of macronutrients and osmolarity would be the most appropriate depending on the characteristics of the endurance test, as a consequence of the large heterogeneity in the samples, tests used to generate exhaustion, and exogenous factors used to affect performance and perceived fatigability. Therefore, future research is needed to investigate these issues further.

## 5. Conclusions

Exogenous factors related to nutrition and hydration strategies can affect performance and perceived fatigability in endurance tests lasting between 45 min and 3 h. More specifically, CHO intake at 6–8% during an endurance test could increase the time to exhaustion. CHO intake as an exogenous factor may also be effective in decreasing the HR. With respect to the use of CHO + PROT as an exogenous factor during an endurance test, a reduction in lactate accumulation both during and after the test was found. Regarding the use of hydration/dehydration as an exogenous factor to modify the onset of fatigue in endurance tests, it was observed that dehydration or inadequate fluid replacement increases body temperature, fatigue, body mass loss, and RPE.

However, exposure to different environmental conditions as exogenous factors to modify performance and perceived fatigability variables were not shown to have as much influence. Thus, endurance tests in hot environments (35–40 °C) resulted in increased skin temperature, decreased temperature gradient, increased HR, and RPE. In contrast, performing the endurance test in low temperature environments or at altitudes ranging from 2000 to 3000 m did not affect the lactate levels, HR, or RPE, respectively, and, therefore, may not significantly affect fatigue.

## Figures and Tables

**Table 1:** Characteristics of the participants and the research designs in the articles included in the systematic review and meta-analysis, related to the use of nutritional and hydration strategies as exogenous factors influencing fatigue.

Authors	Sex (n)	Age (X ± DE)	Characteristics of the Sample	Exogenous Factors	Test Performed	Control Group	Experimental Group	Dependent Variable
Powers et al. [7]	CG: Men 9; EG: Men 9	GC 25.9 ± 5.8; GE 25.9 ± 5.8	Cyclists, highly trained	CHO	Cycling 110% of ventilatory threshold (range 82–88%) to fatigue	Water with placebo	Intake of CHO 7% with electrolytes	Time until exhaustion (min)
Davis et al. [8]	CG: Men 9, Women 7; EG Men 9, Women 7	GC Male 25.8 ± 3.3, Female 22.3 ± 2.6; GE Male 25.8 ± 3.3, Female 22.3 ± 2.6	Healthy, physically active	CHO	1 min of cycling on an ergometer bike at 120–130% of VO_2_max, separated by 3 min of rest until fatigue	Before 4 mL/kg body mass of flavored drink, for 4 mL/kg body mass of flavored drink every 20 min	Before 4 mL/kg body mass of 18% CHO drink, during 4 mL/kg body mass of 6% CHO drink every 20 min	Time until exhaustion (min)
Fraga et al. [9]	CG Men 6; EG Men 6	GC 26 ± 6; GE 26 ± 6	Well-trained runners	CHO	Participants ran to fatigue at 85% of VO_2_max	Ingestion or mouth rinse with a placebo drink without CHO	Ingestion of a 6% CHO solution or mouth rinse with an 8% CHO solution	Time until exhaustion (min), lactate (mmol/L), HR (bpm)
Coyle et al. [10]	CG Men 9, Women 1; CG Men 9, Women 1	CG 28 ± 2; EG 28 ± 2	Trained cyclists	CHO	74 ± 2% VO_2_ max (range 70–79% of VO_2_max until fatigue)	A placebo	At 20 min 1 g/kg body mass, at 60, 90, 120 min 0.25 g/kg body mass t or 6% of drink with CHO	Time until exhaustion (min), lactate (mmol/L)
Nassis et al. [11]	CG Men 8, Women 1; EG Men 8, Women 1	CG 25 ± 4.3; EG 25 ± 4.3	Trained runners (women and men)	CHO	Repeated sets of 15 s of fast running (at 80% of VO_2_max for the first 60 min, at 85% of VO_2_max from 60 to 100 min of exercise, and, finally, at 90% of VO_2_max from 100 min of exercise to exhaustion), separated by 10 s of slow running (at 45% VO_2_max)	Water placebo	6.9% CHO and electrolytes immediately before the race (3 mL/kg body mass) and every 20 min thereafter (2 mL/kg body mass)	Time until exhaustion (min)
Hornery et al. [12]	CG Men 8; EG Men 8	CG 18.3 ± 3; EG 18.3 ± 3	Highly trained tennis players	CHO	Simulated tennis matches (2 h, 40 min)	Placebo was a flavored drink	Caffeine supplementation (3 mg/kg), CHO supplementation (6% solution)	Body mass (kg)
Lee et al. [13]	CG Men 1, Women 5; EG Men 2, Women 4	CG 45.2 ± 10.2; EG 45.2 ± 10.2	Recreational athletes (women and men)	CHO	Cycling exercise with 2 sessions of 45 min	Placebo was a flavored drink	Beverage with 7.5% CHO solution	Body mass (kg)
Gomes et al. [14]	CG Men 12; EG Men 12	CG 18 ± 1; EG 18 ± 1	Well-trained tennis players	CHO	Simulated tennis matches (3 h)	Placebo was a flavored water drink	6% CHO maltodextrin drink	Body mass (kg)
Coyle et al. [15]	CG Men 7; EG Men 7	CG 28 ± 1; EG 28 ± 1	Endurance-trained cyclists	CHO	71 ± 1% of VO_2_max until fatigue	Placebo, flavored drink 4 mL/kg body mass	Glucose polymer solution (i.e., 2.0 g /kg at 20 min and 0.4 g/kg every 20 min thereafter)	Lactate (mmol/L), HR (ppm)
Ball et al. [16]	CG Men 8; EG Men 8	CG 26.9 ± 4.4; EG 26.9 ± 4.4	Trained cyclists	CHO	50 min time trial, subjects immediately performed a Wingate anaerobic power test	Flavored drink without CHO or electrolytes	Drink with CHO + electrolytes at 7%	HR (ppm)
Byrne et al. [17]	CG Men 14; EG Men 14	CG 20.7 ± 0.8; EG 20.7 ± 0.8	Soldiers in the Singapore Armed Forces	CHO	Exercise loaded with 14 kg of body mass, consisting of 3 cycles of 60 min of walking at 4.4 km/h and at a 5% gradient, separated by 15 min of seated rest, carried out in an environmental chamber (35 °C ambient temperature, 55% relative humidity, 2 m s^−1^ wind speed, and 600 W solar radiation)	Placebo	5.8 g CHO, 46 mg sodium and 13 mg potassium per 100 mL every 15 min	HR (ppm)
Van Essen et al. [18]	CG Men 10; EG Men 10	CG 24 ± 2; EG 24 ± 2	Trained male cyclists	CHO + PROT	80 km laboratory time trial	Placebo	2% protein added to a 6% CHO beverage	Lactate (mmol/L), insulin in blood (µL/mL)
Hsu et al. [19]	CG Men 14; EG Men 14	CG 23.4 ± 0.8; EG 23.4 ± 0.8	Healthy men	CHO + PROT	5 min warm-up at 55% VO_2_max, then increased to a pace equivalent to 75% VO_2_max, for 30 min. After 30 min, the intensity (incline) was gradually increased by 1% every minute until exhaustion was reached	200 mL H_2_O citrus-flavored drink with 10 mg sweetener	200 mL BCAA drink containing valine (0.5 g), leucine (1 g), isoleucine (0.5 g), arginine (0.5 g), CHO (12.1 g), flavors and sweeteners in 100 mL H_2_O	Lactate (mmol/L), insulin in blood (µL/mL), HR (ppm)
Alghannam et al. [20]	CG Men 6; EG Men 6	CG 26 ± 2; EG 26 ± 2	Healthy men used to running	CHO + PROT	Running on a treadmill at 70% of VO_2_max, until fatigue	0.8 g/kg/h sucrose + 0.4 g/kg/h whey protein hydrolysate	1.2 g/kg/h CHO + 0.4 g/kg/h whey protein hydrolysate	Lactate (mmol/L), HR (ppm)
Valentine et al. [21]	CG Men 11; EG Men 11	CG 20.8 ± 2.4; EG 20.8 ± 2.4	Healthy male cyclists (4 days aerobic training/week, including 2 days cycling training, VO_2_max greater than 45 mL-kg^−1^-min^−1^, and 2 days cycling training, VO_2_max greater than 45 mL-kg^−1^-min^−1^)	CHO + PROT	75% of VO_2_max until exhaustion	250 mL placebo	CHO + Pro (7.75%/1.94%)	Lactate (mmol/L), HR (ppm), RPE
Bastos-Silva et al. [22]	CG Men 13; EG Men 13	CG 23.1 ± 2.6; EG 23.1 ± 2.6	Healthy men	CHO + PROT	Participants pedaled at 80% of their respiratory compensation point and 110% of their maximum power up to the point of exhaustion	Mouthwash with a control drink	Carbohydrate mouthwash	RPE
Javierre et al. [23]	CG Men 20; EG Men 20	CG 21.3 ± 0.7; CG 21.3 ± 0.7	Healthy young people	CHO + PROT	Submaximal exercise on a cycloergometer, 50 of their respective VO_2_max for 10 min, followed by maximal intensity exercise for 30 s. This sequence was repeated three times, and after the fourth set, each participant continued to exercise at the highest speed they could sustain for 20 min	2/day 500 mL of an isotonic beverage containing 2.4% glucose and fructose mixture (50% each) and 45 mOsmol/L of Na+ and 7 mOsmol/L of K+; along with the beverage, a capsule containing a placebo	2/day 500 mL of an isotonic beverage containing 2.4% glucose and fructose mixture (50% each) and 45 mOsmol/L of Na+ and 7 mOsmol/L of K+; together with the drink, a capsule containing 300 mg of L-tryptophan (“active” compound)	HR (bpm), RPE
Baker et al. [38]	CG Men 17; EG Men 17	CG 21.1 ± 2.4; EG 21.1 ± 2.4	Basketball players	Hydration	Walking (50% VO_2_max) in the heat (40 °C and 20% relative humidity), plus a simulated basketball match	40 °C and 20% relative humidity, 1% dehydrated	40 °C and 20% relative humidity, euhydrated	RPE
Moyen et al. [39]	CG Men 103, Women 16; EG: Men 103, Women 16	CG 46 ± 9; EG 46 ± 9	Trained cyclists	Hydration	161 km of endurance cycling in Wichita Falls, Texas	Dehydrated (USG ≥ 1022) in a thermoneutral environment (23 °C)	Euhydrate (USG ≤ 1018) in a thermoneutral environment (23 °C)	RPE
Hillman et al. [40]	CG Men 7; EG Men 7	CG 36.6 ± 6; EG 36 ± 6	Healthy, trained cyclists (power output (W) at lactate threshold (LT): 199 ± 19 W)	Hydration	90 min cycling exercise at 95% lactic threshold followed by a 5 km time trial in 4 attempts	Dehydrated in a thermoneutral environment (23 °C)	Euhydrated in a thermoneutral environment (23 °C)	RPE
Linseman et al. [42]	CG Men 14; EG Men 14	CG 21.3 ± 0.2; EG 21.3 ± 0.2	Expert male hockey players	Hydration	70 min ice hockey match	Dehydration of ~2% BM without liquid	Hydration with CHO and electrolyte solution	Body mass (kg)
Hillman et al. [43]	CG Men 7; EG Men 7	CG 28 ± 8; EG 28 ± 8	Men trained in cycling	Hydration	Cycling time trial of 90 min	No liquid	26 mL-kg^−1^ BM water	Body mass (kg)
Wittbrodt et al. [44]	CG Men 12; EG Men 12	CG 22.2 ± 2.4; EG 22.2 ± 2.4	Recreationally active men	Hydration	50 min of cycling (60% VO_2_max) in the heat (32 °C; 65% RH)	No liquid	Ad libitum with water	Body mass (kg)

CG: control group; EG: experimental group; CHO: carbohydrate; CHO + PROT: carbohydrate with protein; HR: heat rate; RPE: rate of perceived exertion; USG: urine specific gravity.

**Table 2:** Characteristics of the participants and the research designs in the articles included in the systematic review and meta-analysis, related to exposure to environmental factors as exogenous factors influencing fatigue.

Authors	Sex (n)	Age (X ± DE)	Characteristics of the Sample	Exogenous Factors	Test Performed	Control Group	Experimental Group	Dependent Variable
Wadley et al. [63]	CG Men 12; EG Men 12	CG 28 ± 4; EG 28 ± 4	Endurance-trained cyclists	Height	Cycling 75 min at 70% of VO_2_max to fatigue	Normoxia	Hypobaric hypoxia, equivalent to 2000 m above sea level	HR (bpm), RPE
Bradbury et al. [64]	CG Men 12; EG Men 12	CG 26 ± 6; EG 26 ± 6	Trained healthy males	Height	30 min of steady state cycling exercise (50% VO_2_max), followed by 15 min of exercise at own pace	Thermoneutral and normoxia (250 m, 20 °C, 30–50% relative humidity (rh))	Warm hypobaric hypoxia (HH; 3000 m, 35 °C, 30% rh)	HR (bpm), RPE
Komiyama et al. [65]	CG Men 16; EG Men 16	CG 23 ± 2.3; EG 23 ± 2.3	Healthy and physically active males	Height	Participants cycled on an ergometer for 30 min in normoxia and moderate hypoxia, while maintaining their heart rate (HR) at 140 beats/min	Normoxia	Hypoxia (fraction of inspired oxygen (FIO2) = 0.15, corresponding to an altitude of approx. 2600 m)	HR (bpm), RPE
Ansley et al. (2008) [66]	CG Men 9; EG Men 9	CG 24 ± 7; EG 24 ± 7	Acclimatized healthy and recreationally active	Cold	75% of VO_2_max until fatigue using a cycloergometer at an ambient temperature of 29 ± 1.0 °C with a relative humidity of approximately 50%	No head cooling	With head cooling	Lactate (mmol/L)
Ferguson et al. [67]	CG Men 12; EG Men 12	CG 29 ± 8; EG 29 ± 8	Healthy and trained cyclists	Cold	15 km time trial in different environmental conditions	Exercise at 23 °C	Exercise at 0 °C	Lactate (mmol/L)
Ito et al. [68]	CG Men 12; EG Men 12	CG 21 ± 7; EG 21 ± 7	Healthy men	Cold	Treadmill at 80% VO_2_max	No rain	With rain at 5 °C	Lactate (mmol/L)
VanHaitsma et al. [46]	CG Men 20; EG Men 20	CG 36.1 ± 9.7; EG 36.1 ± 9.7	Trained cyclists	Heat	Time trial of 40 km with a “race” effort	21 °C and a relative humidity of 20%	35 °C and a relative humidity of 25%	HR (bpm), RPE
Wright et al. [47]	CG Men 7, Women 2; EG Men 7, Women 2	CG 26 ± 2.5; EG 26 ± 2.5	Healthy resistance-trained or not resistance trained	Heat	Participants walked on a treadmill (4.5 km h^−1^, 2% incline) in a warm and dry environment (40 °C, 30% relative humidity, wind speed\0.1 m s^−1^)	Untrained at 40 °C, 30% relative humidity	Trained at 40 °C, 30% relative humidity	RPE
Périard et al. [48]	CG Men 8; EG Men 8	CG 29.9 ± 8.1; EG 26.3 ± 4.4	Endurance-trained (>250 km/s) male cyclists not acclimatized to heat or untrained	Heat	Ergometer biking, started pedaling at 150 W for 3 min to “warm up”, 60% of VO_2_max (222.1 + 26.5 W), until exhaustion	Untrained, at 60% VO_2_max, 40 °C. Untrained at 75% VO_2_max, 40 °C	Trained, at 60% VO_2_max, 40 °C. Trained, at 75% VO_2_max, 40 °C	RPE, HR (bpm), skin temperature (°C), temperature gradient (°C)
Wingo et al. [49]	CG Men 9; EG Men 9	CG 25 ± 4; EG 25 ± 4	Trained cyclists	Heat	35 °C, 40% relative humidity, 15 min cycling at 60% VO_2_max, plus 45 min cycling at 60% VO_2_max	With variable HR	HR held constant	RPE, HR (bpm)
Low et al. [50]	CG Men 9; EG Men 9	CG 25.11 ± 3.55; EG 25.11 ± 3.55	Active men	Heat	∼60% of VO_2_max at 70–80 rpm for 45 min on an ergometer bike	Exercise at 18 °C	Exercise at 30 °C	HR (bpm), skin temperature (°C), temperature gradient (°C)

CG: control group; EG: experimental group; HR: heat rate; RPE: rate of perceived exertion.

**Table 3:** Quality analysis of the articles included in the systematic review and meta-analysis according to the PEDro scale criteria.

Authors	*n.1* Specified Eligibility Criteria	*n.2* Random Allocation	*n.3* Concealed Allocation	*n.4* Groups Similar at Baseline	*n.5* Participants Blinding	*n.6* Intervention Blinding	*n.7* Measurer Blinding	*n.8* Less Than 15% Dropouts	*n.9* Intention-to-Treat Analysis	*n.10* Between-Group Statistical Comparisons	*n.11* Point Measures and Variability Data	Total
Nutritional and hydration strategies as exogenous factors												
CHO												
Powers et al. [7]	NO	YES	YES	YES	YES	YES	YES	YES	YES	YES	YES	10
Davis et al. [8]	YES	YES	YES	YES	YES	YES	YES	YES	YES	YES	YES	11
Fraga et al. [9]	YES	YES	YES	YES	YES	YES	YES	YES	YES	YES	YES	11
Coyle et al. [10]	YES	YES	YES	YES	YES	YES	YES	YES	YES	YES	YES	11
Nassis et al. [11]	YES	YES	YES	YES	YES	YES	YES	YES	YES	YES	YES	11
Hornery et al. [12]	YES	YES	YES	YES	YES	NO	NO	YES	YES	YES	YES	9
Lee et al. [13]	YES	YES	YES	YES	YES	NO	NO	YES	YES	YES	YES	10
Gomes et al. [14]	YES	YES	YES	YES	YES	YES	YES	YES	YES	YES	YES	11
Coyle et al. [15]	YES	NO	NO	YES	NO	NO	NO	YES	YES	YES	YES	6
Ball et al. [16]	YES	NO	YES	YES	NO	NO	NO	YES	YES	YES	YES	7
Byrne et al. [17]	YES	YES	YES	YES	NO	NO	NO	YES	YES	YES	YES	8
CHO + PROT												
Van Essen et al. [18]	YES	YES	YES	YES	YES	YES	YES	YES	YES	YES	YES	11
Hsu et al. [19]	YES	YES	YES	YES	YES	YES	YES	YES	YES	YES	YES	11
Alghannam et al. [20]	YES	YES	YES	YES	YES	YES	YES	NO	YES	NO	NO	8
Valentine et al. [21]	YES	NO	NO	YES	YES	YES	YES	YES	YES	YES	YES	9
Bastos-Silva et al. [22]	YES	YES	YES	YES	YES	YES	YES	YES	YES	YES	YES	11
Javierre et al. [23]	YES	YES	YES	YES	YES	YES	YES	YES	YES	YES	YES	11
Hydration												
Baker et al. [38]	YES	YES	YES	YES	YES	YES	YES	YES	YES	YES	YES	11
Moyen et al. [39]	YES	NO	NO	YES	NO	NO	NO	YES	YES	YES	YES	6
Hillman et al. [40]	YES	YES	YES	YES	NO	NO	NO	YES	YES	YES	YES	8
Linseman et al. [42]	YES	YES	NO	YES	NO	NO	NO	YES	YES	YES	YES	7
Hillman et al. [43]	YES	NO	NO	YES	NO	NO	NO	YES	YES	YES	YES	6
Wittbrodt et al. [44]	NO	YES	NO	YES	NO	NO	NO	YES	YES	YES	YES	6
Environmental conditions as exogenous factors												
Altitude												
Wadley et al. [63]	YES	NO	NO	YES	NO	NO	NO	YES	YES	YES	YES	6
Bradbury et al. [64]	NO	YES	YES	NO	YES	NO	NO	YES	YES	YES	YES	7
Komiyama et al. [65]	YES	YES	YES	YES	YES	NO	NO	YES	YES	YES	YES	9
Cold												
Ansley et al. (2008) [66]	NO	NO	NO	NO	NO	NO	NO	YES	YES	YES	YES	4
Ferguson et al. [67]	YES	YES	YES	YES	YES	YES	YES	YES	YES	YES	YES	11
Ito et al. [68]	NO	NO	NO	YES	NO	NO	NO	YES	YES	YES	YES	5
Heat												
VanHaitsma et al. [46]	YES	NO	NO	YES	NO	NO	NO	YES	YES	YES	YES	6
Wright et al. [47]	YES	NO	NO	NO	NO	NO	NO	YES	YES	YES	YES	5
Périard et al. [48]	YES	YES	NO	NO	NO	NO	NO	YES	YES	YES	YES	6
Wingo et al. [49]	YES	NO	NO	YES	NO	NO	NO	YES	YES	YES	YES	6
Low et al. [50]	NO	NO	NO	YES	NO	NO	NO	YES	YES	YES	YES	5

**Table 4:** Meta-analysis results in relation to nutrient and fluid intake.

	Dependent Variable	Comparison	Number of Studies	Number of Experimental Group (Total n)	Number of Control Group (Total n)	SMD	95% CI o IC	z	*p*	Heterogeneity	*I* ^2^	Rosenthal Tolerance Index
CHO during the test	Time until exhaustion (min)	EG post-test vs. CG post-test	5	5 (*n* = 41)	5 (*n* = 50)	1.46	0.96 to 1.97	5.57	<0.001	Q = 26.56 (*p* <0.001; gl = 4)	85% (95%CI = 67; 93%)	67
	Body mass (kg)	Diff EG pre-post vs. diff CG pre-post	3	3 (*n* = 30)	3 (*n* = 30)	0.01	−0.49 to 0.51	0.05	0.959	Q = 0.02 (*p* = 0.988; gl = 2)	−8697% (95%CI = 0; −815%)	−2.97
	Lactate (mmol/L)	Diff EG pre-test—intermediate test 1 (15–30′) vs. diff CG pre-test—intermediate test 1 (15–30′)	3	3 (*n* = 23)	3 (*n* = 23)	0.31	−0.28 to 0.91	1.03	0.303	Q = 3.63 (*p* = 0.163; gl = 2)	45% (95%CI = 0; 84%)	1.66
	Lactate (mmol/L)	Diff EG intermediate test 1 (15–30′)—intermediate test 2 (30–60′) vs. CG intermediate test 1 (15–30′)—intermediate test 2 (30–60′)	3	3 (*n* = 23)	3 (*n* = 23)	0.18	−0.57 to 0.94	0.48	0.633	Q = 3.19 (*p* = 0.203; gl = 2)	37% (95%CI = 0; 80%)	−0.82
	HR (ppm)	Diff EG intermediate test 1 (15–30′)—intermediate test 2 (30–60′) vs. CG intermediate test 1 (15–30′)—intermediate test 2 (30–60′)	4	4 (*n* = 35)	4 (*n* = 35)	−0.59	−1.07 to −0.09	2.36	0.018	Q = 3.97 (*p* = 0.264; gl = 3)	25% (95%CI = 0; 88%)	2.78
CHO + PROT during the test	Lactate (mmol/L)	EG intermediate test (30–60′) vs. CG intermediate test (30–60′)	4	4 (*n* = 41)	4 (*n* = 41)	−0.47	−0.92 to −0.02	2.06	0.039	Q = 5.31 (*p* = 0.150; gl = 3)	44% (95%CI = 0; 81%)	4.70
	Lactate (mmol/L)	EG post-test vs. CG post-test	3	3 (*n* = 31)	3 (*n* = 31)	0.02	−0.48 to −0.52	0.07	0.946	Q = 0.54 (*p* = 0.762; gl = 2)	−268% (95%CI = 0; 62%)	−2.49
	RPE	EG intermediate test (15–20′) vs. intermediate test (15–20′)	3	3 (*n* = 44)	3 (*n* = 44)	−0.75	−1.91 to 0.41	1.27	0.204	Q = 12.92 (*p* = 0.002; gl = 2)	85% (95%CI = 54%; 95%)	12.83
	HR (ppm)	EG intermediate test (15–45′) vs. intermediate test (15–45′)	4	4 (*n* = 51)	4 (*n* = 51)	−0.06	−0.45 to 0.33	0.31	0.756	Q = 0.305 (*p* = 0.959; gl = 3)	−882% (95%CI = 0; −50%)	−3.56
	HR (ppm)	EG post-test vs. CG post-test	3	3 (*n* = 37)	3 (*n* = 37)	0.11	−0.35 to 0.56	0.46	0.647	Q = 0.259 (*p* = 0.879; gl = 2)	−671% (95%CI = 0; 20%)	−2.65
Hydration	RPE	EG post-test vs. CG post-test	3	3 (*n* = 143)	3 (*n* = 143)	0.29	0.05 to 0. 52	2.40	0.016	Q = 0.492 (*p* = 0.782; gl = 2)	−307% (95%CI = 0; 58%)	3.15
	Body mass loss (kg)	EG post-test vs. CG post-test	3	3 (*n* = 33)	3 (*n* = 33)	0.62	0.11 to 1.14	2.37	0.018	Q = 9.539 (*p* = 0.008; gl = 2)	79% (95%CI = 33%; 93%)	10.76

CG: control group; EG: experimental group; Diff: differences; CHO: carbohydrate; CHO + PROT: carbohydrate with protein; HR: heat rate; RPE: rate of perceived exertion.

**Table 5:** Meta-analysis results in relation to the environmental conditions.

	Dependent Variable	Comparison	Number of Studies	Number of Experimental Group (Total n)	Number of Control Group (Total n)	SMD	95% CI o IC	z	*p*	Heterogeneity	*I* ^2^	Rosenthal Tolerance Index
Altitude	RPE	EG post-test vs. CG post-test	3	3 (*n* = 40)	3 (*n* = 40)	0.03	−0.41 to 0.47	0.13	0.894	Q = 0.85 (*p* = 0.653; gl = 2)	−135% (95%CI = 0; 76%)	−2.40
	HR (ppm)	EG post-test vs. CG post-test	3	3 (*n* = 40)	3 (*n* = 40)	0.03	−0.41 to 0.47	0.14	0.890	Q = 0.244 (*p* = 0.885; gl = 2)	−720% (95%CI = 0; 15%)	−2.82
Cold	Lactate (mmol/L)	EG post-test vs. CG post-test	3	3 (*n* = 33)	3 (*n* = 33)	−0.07	−0.60 to 0.46	0.27	0.790	Q = 19.932 (*p* = 0.005; gl = 2)	90% (95%CI = 73%; 96%)	16.84
Heat	RPE	EG post-test vs. CG post-test	3	3 (*n* = 70)	3 (*n* = 70)	2.18	1.67 to 2.68	8.40	<0.001	Q = 71.080 (*p* < 0.001; gl = 6)	92% (95%CI = 95%; 85%)	340.09
	HR (ppm)	EG post-test vs. CG post-test	4	4 (*n* = 70)	4 (*n* = 70)	1.13	0.72 to 1.54	5.41	<0.001	Q = 55.507 (*p* < 0.001; gl = 6)	89% (95%CI = 80%; 94%)	160.08
	Skin temperature (°C)	EG post-test vs. CG post-test	3	3 (*n* = 41)	3 (*n* = 41)	12.37	4.40 to 20.33	3.04	0.002	Q = 72.51 (*p* < 0.001; gl = 4)	94% (95%CI = 90%; 97%)	208.26
	Temperature gradient (°C)	EG post-test vs. CG post-test	3	3 (*n* = 41)	3 (*n* = 41)	−3.78	−4.81 to −2.75	7.20	<0.001	Q = 66.418 (*p* < 0.001; gl = 4)	89% (95%CI = 97%; 89%)	207.92

CG: control group; EG: experimental group; Diff: differences; HR: heat rate; RPE: rate of perceived exertion.

## Data Availability

The data presented in this article are available on request from the corresponding author.

## Supplementary Materials

The following supporting information can be downloaded at: https://www.mdpi.com/article/10.3390/nu15122700/s1, File S1: Search formula used in the systematic review.
