# Supplementary material for: Influence of Exogenous Factors Related to Nutritional and Hydration Strategies and Environmental Conditions on Fatigue in Endurance Sports: A Systematic Review with Meta-Analysis
Source: Nutrients. 2023 Jun 9;15(12):2700. doi: 10.3390/nu15122700 (PMC10305101; doi:10.3390/nu15122700)
Supplement: Supplementary file 1 [file nutrients-15-02700-s001.zip › nutrients-2415711-supplementary.pdf]

Supplementary S1. Search formula used in the systematic review.
(fatigue OR onset of fatigue OR start of fatigue OR beginning of fatigue OR Decreased performance OR Decrease in performance OR Performance decline OR Acute fatigue OR Chronic fatigue OR Central fatigue OR Peripheral fatigue OR Mind Charge OR Mental load OR Mental fatigue) and (endurance sports OR endurance sport OR endurance OR triathlon OR cycling OR ultra-marathon OR ultramarathon OR marathon OR wild Swimming OR open water swimming OR Ironman OR Ironman triathlon OR athlete) and (young OR adult OR adults) and (Fatigue factors OR Factors of Fatigue OR Fatigue factor OR Factor of Fatigue OR Psychological aspects OR emotional state OR Psychology OR Psychological profile OR stress OR Stressful situations OR Stressing situations OR anxiety OR poms vic OR perceived range of effort OR range of perceived effort OR perceived stress range OR range of perceived stress OR Pain) and (Physiology or physiological parameters OR physiologic parameters OR ammonium OR ammonia OR temperature OR Body temperature OR body's temperature OR Catecholamines OR Catecolamines OR cortisol OR serotonin OR dopamine OR endorphin OR endorphine OR noradrenaline Or norepinephrine OR noradrenalin OR Adrenocorticotropic Hormone OR Testosterone OR Tryptophan OR Triptofan OR Toxicity) and (nutrition OR glycogen OR glucogen OR Glycogen reserves OR Glycogen stores OR glucose OR sugar OR sugars OR Glycolysis OR Glucolysis OR Lipolysis OR Proteolysis OR Muscle catabolism OR Muscular catabolism OR Muscle loss OR Catabolic OR Catabolism OR Muscle destruction OR Muscular destruction OR Proteins OR Metabolism OR Energy expenditure OR Energy metabolism OR Energetic metabolism OR Energy deficit OR Energetic deficit OR Deficit caloric OR Caloric Deficit OR Caloric deficiency OR Carbohydrates OR Carbohydrate OR carbs OR Glucides OR lipids OR fat OR greases OR Fatty acids OR Gramic acids OR Grassy Acids OR Free fatty acids OR Triglycerides OR Amino acids OR Aminoacids OR Amino-acids OR Essential amino acids OR Essential Aminoacids OR BCAA OR Branched-Chain Amino Acids OR Branched Amino Acids OR Branched aminoacids OR Nutritional Strategies OR Nutrition Strategies OR Diet OR Nutritional advice OR Nutritional counseling OR Nutrition counseling OR Relative Energy Deficency in Sport OR RED-S OR triad of the sports woman OR sportswoman triad OR triad of the sports woman OR triad of the female athlete OR athlete's triad OR sportsman's triad OR athlete triad OR sports triad OR Hydration OR Dehydration).
